# Is the mechanism of these supraventricular tachycardias simply explained by reverse rotation?

**DOI:** 10.1002/joa3.12824

**Published:** 2023-02-06

**Authors:** Satoshi Hayashida, Koichi Nagashima, Melvin M. Scheinman, Satoshi Higuchi, Yasuo Okumura

**Affiliations:** ^1^ Division of Cardiology, Department of Medicine Nihon University School of Medicine Tokyo Japan; ^2^ Division of Cardiology, Section of Cardiac Electrophysiology University of California San Francisco San Francisco California USA

**Keywords:** atrioventricular nodal reentrant tachycardia, concealed atrio‐Hisian tract, lower common pathway, supraventricular tachycardia

A 49‐year‐old female without overt structural heart disease underwent an electrophysiological study for a supraventricular tachycardia. Right ventricular extrastimuli delivered at coupling intervals from 440 to 450 ms at a basic pacing cycle length (PCL) of 600 ms revealed the earliest activation in the His bundle region with a lesser decremental property, but those delivered ≤430 ms revealed a slight change in the earliest atrial activation site to the proximal coronary sinus (pCS) without any His‐atrial (HA) jump phenomenon (Figure 1A). Those observations suggested the presence of a dual retrograde atrioventricular nodal (AVN) pathway with a similar conduction velocity and weak retrograde conductivity over the fast pathway. A decrease in the atrial extrastimulus coupling interval revealed an atrio‐Hisian (AH) jump (Figure 1B) indicating a dual AVN pathway. A further decrease in the atrial extrastimulus coupling interval exhibited one echo with the earliest activation in the pCS preceding the His potential, which suggested 2 slow pathways (SPs) and the presence of a lower common pathway (LCP) between the 2 SPs (Figure 1C). Two forms of a narrow QRS tachycardia, varying by the ventriculoatrial interval and cycle length, were inducible by atrial extrastimuli; the AH/HA intervals were 314/60 ms for SVT1 (Figure 2A) and 128/154 ms for SVT2 (Figure 2B). Right ventricular (RV) entrainment pacing yielded a VAV response with a long post‐pacing interval (PPI) for both SVT1 (Figure 2A) and SVT2 (Figure 2C), suggesting an AVN reentrant tachycardia (AVNRT). SVT2 was sustained despite the occurrence of AH block (Figure 2D), indicating the presence of a lower common pathway (LCP). Is the mechanism of these SVTs simply explained by reverse rotation?

**FIGURE 1:**
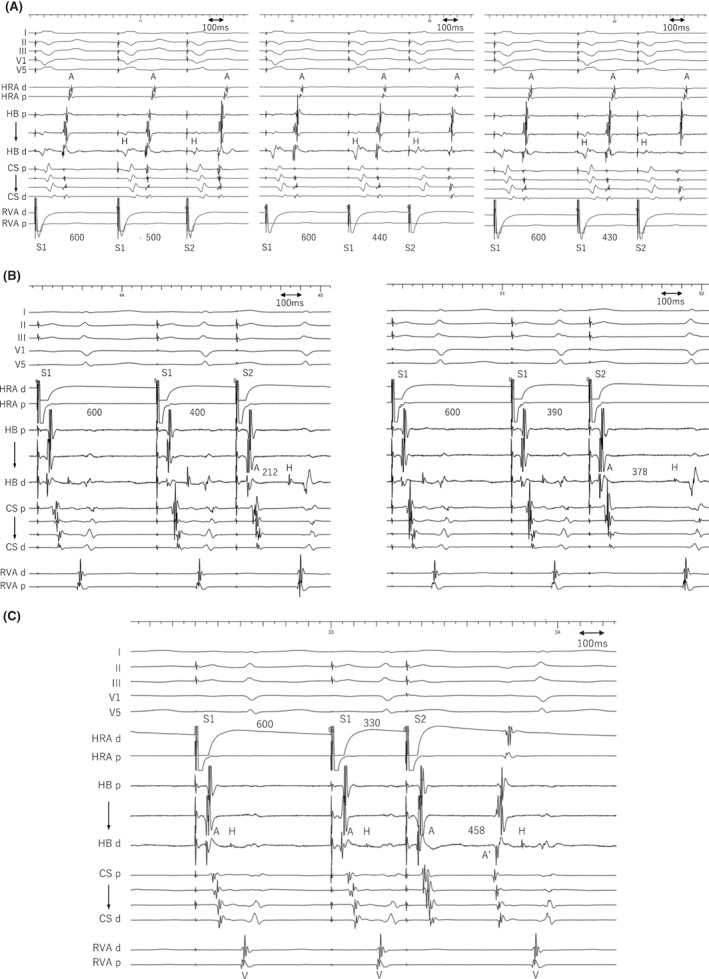
Intracardiac electrograms of ventricular extrastimuli with coupling intervals of 500, 440, and 430 ms at a basic pacing cycle length of 600 ms (A). Decremental ventriculoatrial conduction accompanied by a slight change in the earliest atrial activation site in the His bundle region to the proximal coronary sinus without any His‐atrial jump occurred. Intracardiac electrograms of atrial extrastimuli with coupling intervals of 400, 390 (B), and 330 ms (C) at a basic pacing cycle length of 600 ms. (B) AH jump is observed. (C) An atrial echo (A') is seen preceding the His potential. A, atrial; CS, coronary sinus; d, distal; H, His bundle; HB, His bundle; HRA, high right atrium; p, proximal; RVA, right ventricular apex; S, stimulus; SVT, supraventricular tachycardia; V, ventricle.

**FIGURE 2:**
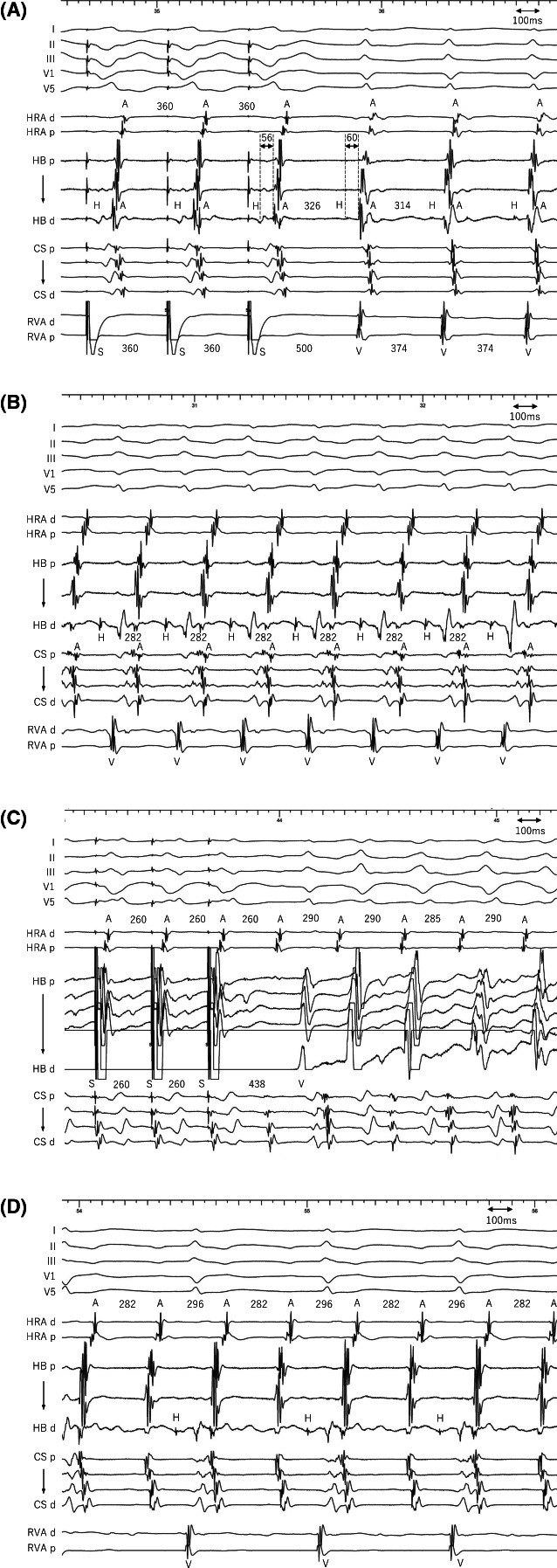
(A) Intracardiac electrograms during right ventricular entrainment pacing during SVT1. The HA interval is slightly shorter during RV entrainment pacing (56 ms) than during the tachycardia (60 ms), indicating the absence of an LCP. Intracardiac electrograms of SVT2 (B), right ventricular entrainment pacing from the para‐Hisian area during SVT2 (C), and SVT2 sustained despite the occurrence of 2:1 atrio‐Hisian block (D). A, atrial; CS, coronary sinus; d, distal; H=His bundle; HB=His bundle; HRA, high right atrium; p, proximal; RVA, right ventricular apex; S, stimulus; SVT, supraventricular tachycardia; V, ventricle.

The differential diagnoses in these tachycardias included atrial tachycardia (AT), orthodromic reciprocating tachycardia (ORT) via an accessory pathway, and AVNRT. SVT1 had a short RP and H‐A‐V pattern with the earliest atrial activation in the His‐bundle region, which excluded ORT (Figure 2A). Slow‐fast AVNRT was diagnosed by a VAV response after RV overdrive pacing, which ruled out AT (Figure 2A). A PPI—tachycardia cycle length (TCL) of 126 ms also supported AVNRT. Of note, the HA interval was slightly shorter during RV entrainment pacing than during the tachycardia, indicating the absence of an LCP.^1^

In contrast, SVT2 had the earliest atrial activation in the pCS region (Figure 2B). AT was ruled out by a VAV response after RV entrainment pacing with a long VA interval (Figure 2C). The SVT sustained with AH block, which was diagnostic for AVNRT with LCP block excluding ORT (Figure 2D). Because the AH interval was <200 ms and HA interval > 70 ms, fast‐slow AVNRT was diagnosed.^2^ The atrial cycle was reproducibly delayed after the beat with ventricular conduction. This reproducible TCL alternans during AH block was possibly as a result of the change in the lower turnround site between the FP and SP; the circuit might have been slightly shortened during the LCP block.

Generally, the mechanism of slow‐fast AVNRT and fast‐slow AVNRT is considered to be reverse rotation in the fast pathway and slow pathway. However, an LCP connecting the fast pathway and slow pathway appears to be present in fast‐slow AVNRT but not in slow‐fast AVNRT.^2^ The most plausible mechanism of this paradoxical observation can be explained by the concealed AH tract (cAHT) described by Otomo et al.^3^ The anterograde limb of fast‐slow AVNRT is the fast pathway, which is reported to always be connected to the LCP,^2^ but the retrograde limb in slow‐fast AVNRT might be the cAHT, which is not connected to the LCP but is to the His bundle (Figure 3).

**FIGURE 3:**
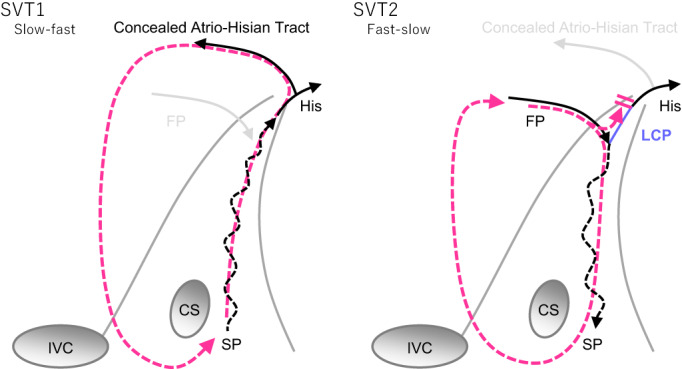
Potential schematic diagrams of the tachycardia circuit of SVT1 (left panel) and SVT2 (right panel). FP, fast pathway; LCP, lower common pathway; SP, slow pathway; SVT, supraventricular tachycardia.

Recently, this old yet new concept was histologically proven by Anderson, et al. as the “last connection” of the fast pathway to the atrial tissue.^4^

The cAHT constituted the retrograde limb of the slow‐fast AVNRT circuit in a setting with the absence of an LCP, which was characterized by the lack of a decremental property or the absence of adenosine triple phosphatase (ATP) sensitivity.^3^ Although the reactivity to ATP was not assessed in this case, the ATP injection test might support our diagnosis of cAHT.

Not a few electrophysiologists would not believe that the circuits of slow‐fast AVNRT and fast‐slow AVNRT are the same but in the reverse direction; so‐called fast‐slow AVNRT is a slow‐slow AVNRT via a leftward inferior extension as the anterograde limb and rightward inferior extension as the retrograde limb.^5^ The basis of that hypothesis is that the HA interval during ventricular pacing at the tachycardia cycle length is much longer than that during fast‐slow AVNRT (compared with the slightly shorter HA interval during ventricular pacing during slow‐fast AVNRT),^5^ which is similar to our observation. Therefore, the concept of a cAHT may be another interpretation to explain the mechanisms why the circuits of slow‐fast AVNRT and fast‐slow AVNRT differ even when those tachycardias are inducible in the same patient.

Based on the current old yet new concept, inducible slow‐fast AVNRT and fast‐slow AVNRT may not necessarily have a reverse rotation relationship, and to the best of our knowledge, this is the first report of a case that has been proven to have the presence of a cAHT by verifying the details of two tachycardias with differential turnaround sites.

A radiofrequency application at the earliest atrial activation site during fast‐slow AVNRT terminated the fast‐slow AVNRT and also rendered the slow‐fast AVNRT non inducible. The mechanism of the induced slow‐fast AVNRT and fast‐slow AVNRT cannot necessarily be explained by reverse rotation but can be by a cAHT as a retrograde limb of the slow‐fast AVNRT.

## FUNDING INFORMATION

This report was not supported by any grant or company.

## CONFLICT OF INTEREST STATEMENT

The authors have no conflict of interest to declare.

## ETHICS APPROVAL STATEMENT

Approval was obtained from the local ethics committee.

## PATIENT CONSENT STATEMENT

The patient has provided consent for publication.

## CLINICAL TRIAL REGISTRATION

None.
